# Taking a Stab at Cancer; Oncolytic Virus-Mediated Anti-Cancer Vaccination Strategies

**DOI:** 10.3390/biomedicines5010003

**Published:** 2017-01-04

**Authors:** Amelia Sadie Aitken, Dominic Guy Roy, Marie-Claude Bourgeois-Daigneault

**Affiliations:** 1 Ottawa Hospital Research Institute, Center for Cancer Therapeutic, Ottawa, ON K1H 8L6, Canada; aaitk100@uottawa.ca (A.S.A.); dominicguyroy@gmail.com (D.G.R.); 2 Department of Biochemistry, Microbiology and Immunology, Ottawa, ON K1H 8M5, Canada

**Keywords:** oncolytic virus, vaccination strategy, cancer, immunotherapy, immunization, tumour-associated antigen, prime-boost, novel therapy

## Abstract

Vaccines have classically been used for disease prevention. Modern clinical vaccines are continuously being developed for both traditional use as well as for new applications. Typically thought of in terms of infectious disease control, vaccination approaches can alternatively be adapted as a cancer therapy. Vaccines targeting cancer antigens can be used to induce anti-tumour immunity and have demonstrated therapeutic efficacy both pre-clinically and clinically. Various approaches now exist and further establish the tremendous potential and adaptability of anti-cancer vaccination. Classical strategies include ex vivo-loaded immune cells, RNA- or DNA-based vaccines and tumour cell lysates. Recent oncolytic virus development has resulted in a surge of novel viruses engineered to induce powerful tumour-specific immune responses. In addition to their use as cancer vaccines, oncolytic viruses have the added benefit of being directly cytolytic to cancer cells and thus promote antigen recognition within a highly immune-stimulating tumour microenvironment. While oncolytic viruses are perfectly equipped for efficient immunization, this complicates their use upon previous exposure. Indeed, the host’s anti-viral counter-attacks often impair multiple-dosing regimens. In this review we will focus on the use of oncolytic viruses for anti-tumour vaccination. We will explore different strategies as well as ways to circumvent some of their limitations.

## 1. Introduction

The field of cancer therapy has seen significant progression within recent years and as a result, has directly improved the outlook for cancer patients. Many cancers respond well to current therapies, with some types having a high rate of long-term remission. Despite these advances, the treatment of late-stage disseminated disease, as well as the frequent occurrence of treatment-resistance, remain challenges that are unmet by current standards of care. The quest for the development of alternative options, particularly for treatment-refractory cancers, has given rise to many novel strategies including immunotherapy. The idea of teaching the immune system to fight one’s own malignancy has undoubtedly revolutionized cancer treatment. With the recent success and excitement that surround immune checkpoint blockade [1], chimeric-antigen receptor T cells [2,3], adoptive cell therapy [4], and anti-cancer vaccines [5], the field of cancer immunotherapy is gaining popularity and is arguably one of the most promising therapeutic approaches to combating the disease [6]. Oncolytic viruses (OV) are exciting immunotherapeutic candidates as they are a novel and versatile treatment option capable of inducing anti-tumour immunity [7,8,9,10]. In recent years, they have been further engineered for anti-cancer vaccination and this strategy is currently being tested clinically. In this review, we will focus on OVs as a platform for anti-cancer vaccination as well as the different strategies using viruses to trigger anti-tumour immunity.

## 2. Prophylactic Vaccines Targeting Oncogenic Viruses; Better Safe Than Sorry

Prophylactic cancer vaccination provides the obvious advantage of preventing initial disease onset but can be challenging as it requires identification of a causal agent. Interestingly, more than 10% of all cancers are the result of infection by oncogenic viruses [11]. Of these, the Epstein-Barr virus is linked to various forms of lymphoma and carcinoma [12], the human T cell lymphotropic virus-1 is associated with acute T cell leukemia [13], the Kaposi’s sarcoma herpes virus causes soft tissue sarcoma [14], the hepatitis B and C viruses (HBV and HCV) increase the risk of developing hepatocellular carcinoma by up to 300-fold [15] and the human papillomavirus (HPV) is the leading cause of cervical intraepithelial neoplasia and various other genital cancers [16,17]. Promisingly, effective vaccines have been designed for two of these oncoviruses: HPV (Gardasil from Merck and Co. and Cervarix by GlaxoSmithKline Biologics) and HBV (Recombivax HB from Merck and Co., Engerix-B from GlaxoSmithKline Biologics and many more) [17,18]. The HPV vaccines target L1 capsid proteins of the most prevalent HPV serotypes. A clinical study with cohorts consisting of sero-negative women showed 95% protection against viral persistence, development of external genital lesions and cervical intraepithelial neoplasia [17]. Similar success has been observed with HBV vaccines for which a number of clinical trials in areas of high prevalence have shown a decrease of up to 75% in the incidence of hepatocellular carcinoma in vaccinated cohorts [18]. The exciting clinical results obtained from HPV and HBV immunization emphasize the potential of cancer vaccines; however, the majority of cancers are diagnosed only once well established. This further supports the need for effective therapeutic vaccines for patients unable to be treated with preventive immunization. Additionally, the cancer vaccines described above target oncogenic pathogens rather than self-antigens. As a result, the protective immunity is mounted against viral epitopes which are ideal candidates for immunization as they are highly specific for virally-induced cancers. On the other hand, most cancers are not initiated by infection and, therefore, lack the presence of these ideal foreign vaccination targets. Effective vaccination targets for cancers not caused by oncogenic viruses are more difficult to identify and select. During the negative selection step of thymic education, autoreactive T cells are depleted to prevent autoimmunity and only a limited number can be found in the periphery. While this clearance step is crucial to prevent self-destruction, it also impairs tumour recognition, as most cancer antigens are aberrantly expressed or modified self-molecules. Breaking this tolerance is one of the main challenges of anti-tumour vaccination and will be discussed further.

## 3. Therapeutic Anti-Cancer Vaccines

Together with the exploitation of vaccination for the prevention of pathogen-induced carcinogenesis, anti-tumour immunity and immune memory can also be harnessed to treat established disease [19]. Therapeutic vaccines target features that are unique characteristics of cancer cells and have the potential to directly control both primary and metastatic lesions as well as minimizing the incidence of relapse [5]. While some immunization strategies include the targeting of tumour-associated carbohydrates and vasculature, most of the current approaches target tumour antigens [5,20]. Tumour-associated antigens (TAA) used for anti-cancer vaccination can either be commonly shared between cohorts of patients or unique to each individual. Among the commonly shared tumour antigens are the cancer testis antigens (CTA). While the expression of CTAs is usually limited to germinal cells in immune-privileged sites like the testis or the brain, tumours often reactivate CTA expression, making them ideal targets for vaccination [21]. For example, the MAGE family of CTAs and NY-ESO-1 are often expressed by breast, ovarian, lung, and melanoma tumours [21]. Given the large number of patients with CTA positive tumours, there is obvious therapeutic potential for the development of vaccines targeting these antigens following clinical validation of CTA expression. The various anti-cancer vaccination trials have been reviewed recently [22] and the results highlight the therapeutic potential of this strategy. For example, a phase III trial investigating the use of interleukin (IL)-2 and gp100 peptide vaccination for melanoma patients demonstrated an improved overall survival (17.8 vs. 11.1 months) [23]. Another example is Sipuleucel-T, an autologous cell vaccine approach that was approved by the United States Food and Drug Administration (FDA) in 2010 for the treatment of castration-resistant prostate cancer patients. The vaccine consists of antigen presenting cells from the patient’s own blood that have been activated ex vivo with a tumor antigen (prostatic acid phosphatase) fused to granulocyte macrophage colony-stimulating factor (GM-CSF). A phase III trial demonstrated a four month prolongation of the median overall survival for the Sipuleucel-T treatment group compared to the placebo group (21.7 vs. 25.8 months) [24]. In some studies, as is the case of a trial investigating the use of an anti-melanoma vaccine in conjunction with IL-2, the patients experienced autoimmune reactions such as vitiligo (7%) and autoimmune thyroiditis (25%) [25]. These results highlight the efficacy of this strategy but also stress the importance of considering the potential development of autoimmunity when developing an anti-cancer vaccine targeting cancer-associated antigens like CTA. While targeting CTA is an appealing strategy, not all cancers express a common cancer antigen. For these patients, a more personalized vaccination approach is necessary. The collection of mutations specific to each cancer is called the mutanome and these mutations can also be targeted for anti-cancer vaccination [26]. Following this idea, the group of Ugur Sahin has demonstrated that vaccination with either peptides or RNA encoding cancer-specific mutations can generate protective anti-tumour immunity [26,27,28]. Exploiting the mutanome for vaccination is an interesting concept with one major caveat being the potential induction of autoimmunity. Further challenges include the requirement for sequencing of each patient’s tumour, prediction of immunogenic antigens and generation of personalized treatment, all of which can augment the cost and extend the time prior to treatment administration. In addition to the direct targeting of a patient’s mutanome, many classical cancer vaccination methods involve the use of cancer cell lysates, which also allows for the immunization against a broad spectrum of tumour antigens. Early studies have demonstrated the benefits of using tumour cell lysate-loaded dendritic cells [29]. Variations of this approach, such as the administration of three-dimensional polymer matrices loaded with tumour lysates together with adjuvants, are still used to date and have proven to be effective in murine melanoma models [30]. Earlier this year, Cheung et al. explored the use of mechanically disrupted cancer cells or vesicular compartments loaded with adjuvants and demonstrated that these “reduced cancer cells” are extremely immunogenic and favour antigen cross-presentation, which results in powerful adaptive immune responses [31]. In addition to tumour antigens, the use of adjuvants improve vaccination as they provide immune-activating signals in the context of antigens while helping to establish a desirable long-term and cancer-specific immunity [32]. For example, aluminum salts, the most common adjuvant used for human vaccination, induce production of IL-4, which polarizes the immune response towards Th2 rather than Th1 [33]. The opposite effect is obtained when using adjuvants that stimulate pattern recognition receptors. These adjuvants include the viral RNA mimic polyI:C or bacterial flagellin which stimulate toll-like receptor (TLR) 3 and 5, respectively, and naturally induce a pro-inflammatory Th1 response [34]. Following this same concept, viruses can themselves be used directly as adjuvants for vaccination and will be our focus from here on.

## 4. A Viral Strategy

Of the many factors that are believed to be important for the development of efficient anti-tumour immunity, type I interferons are essential for various treatment strategies to reach their full potential. This includes some adjuvants, chemotherapeutic agents, and oncolytic viruses, which require interferons for maximal induction of anti-tumour immunity. A recent review by Zitvogel and colleagues urges tumour immunologists to take “advantage of a sophisticated defence system that originally evolved to clear virus-infected cells” and “dedicate substantial efforts to inducing a state that **mimics viral infection**, featuring the secretion of type I IFNs, in malignant tissues” [35]. This supports the use of viral mimics or viruses themselves to stimulate immunity and constitutes a promising strategy to exploit the pre-existing self-defense mechanisms of the host. The concept of using viruses or viral components therapeutically has been validated in several different experimental settings. An interesting strategy is the use of liposomes, which can be targeted to cancer cells through fusogenic viral proteins. These so-called virosomes have intrinsic anti-tumour activities. Indeed, virosomes based on the hemagglutinating virus of Japan were shown by various groups to activate natural killer and cytotoxic T cells and trigger the secretion of chemokines that attract dendritic cells and T cells to the tumour site [36,37]. Virosomes provide the additional advantage of being designed to allow targeted delivery of DNA, RNA, siRNA, or proteins to induce inflammation and anti-tumour immunity [38]. Following this idea, it has been previously demonstrated that antigen-loaded liposomes coated with the fusogenic hemagglutinin protein from influenza virus induce an improved cytotoxic T lymphocyte response compared to antigen alone [39]. Alternatively, virus-like particles (VLPs) without viral genomes can efficiently be used for vaccination against the virus themselves [40]. This approach has an improved safety profile compared to the use of live virus given that the viruses cannot produce progeny. Interestingly, VLPs can be engineered to specifically target tumour cells. For example, hepatitis E VLPs bearing a ligand that binds to breast cancer cells were shown to target breast tissues in vivo [41]. Therefore, VLPs can be used to deliver therapeutic cargoes to tumours. Similarly, Rous sarcoma VLPs could deliver drugs to colon carcinoma cells [42] and rabbit hemorrhagic disease virus VLPs could deliver gp33 peptide and inhibit the growth of Lewis’ lung carcinoma tumours [43]. Although virosomes and VLPs have proven to be successful vaccines with a potentially improved safety profile, they might not be suitable for all patients as some cancers evolved mechanisms that render them refractory to immunization strategies. The use of live viruses for anti-cancer vaccination is an attractive alternative as these biotherapeutics are self-amplifying agents with the potential of inducing powerful immune responses. The capacity to infect and kill tumour cells is an aspect that makes oncolytic viruses (OV) top-choice candidates for cancer immunotherapy.

## 5. Vaccination Strategies Using Oncolytic Viruses (OVs)

Replicating OVs are effective cancer therapeutics based on their ability to directly target and eliminate cancer cells [44]. Although OV efficacy was initially attributed to the direct killing of tumour cells, it is now well-accepted that OVs themselves can induce anti-tumour immunity. While most of the studies evaluating OV-induced tumour-specific immunity focus on the cellular response, humoral immunity can play an important role as well. Indeed, the binding of tumour-specific antibodies to cancer cells can trigger complement-dependent and antibody-dependent cellular cytotoxicity [45]. Interestingly, it was demonstrated that patients and rabbits treated with oncolytic vaccinia virus developed cancer-specific antibodies that could mediate complement-dependent cytotoxicity [46]. The tumour microenvironment is often characterized by a lack of tumour-reactive immune cells and combined with the tumour itself initiating a number of immunosuppressive mechanisms, the anti-tumour immune response often falls short [47]. Indeed, different tumours can have various degrees of inflammation, which may influence the potential for success of certain immunotherapies. Inflamed tumours are those that are infiltrated by tumour-specific immune cells and, therefore, are particularly good responders to immune checkpoint therapy since the blockade of local suppression revitalizes T cell function. An intermediate phenotype known as “immune exclusion” is characterized by anti-tumour immune cells present at the tumour periphery where they cannot infiltrate. This limits their contact with cancer cells and, thus, impairs killing. Finally, some tumours are categorized as “immunologically ignorant”. For these non-inflamed tumours, immune cells are completely absent, and also referred to as an immune desert [48]. Interestingly, OVs have the capability to revert a non-inflamed phenotype into an inflamed phenotype. Indeed, the release of cancer antigens upon oncolysis in the context of OV-associated molecular patterns such as viral capsid constituents, proteins, viral RNA and DNA stimulate Toll-like receptors and provide the danger signal necessary to initiate innate and adaptive immunity [49]. Additionally, OVs have been shown to trigger immunogenic cell death. As opposed to cells undergoing a “silent” type of death like apoptosis, cells undergoing immunogenic cell death alarm the immune system by increasing surface expression of calreticulin and producing ATP and HMGB1, which trigger immune activation and initiate anti-tumor immunity. The induction of immunogenic cell death by OVs has been demonstrated extensively in vitro as well as in pre-clinical murine models with several OVs including adenovirus (Ad) [50], measles virus [51], Herpes simplex virus (HSV) [10], and coxsackievirus [52]. Interestingly, immunogenic cell death has also been observed clinically in chemotherapy-refractory patients treated with an oncolytic Ad expressing CD40 ligand. For these patients, enhanced survival and disease control correlated with both increased anti-tumour T cell responses and enhanced serum levels of HMGB1, a marker of immunogenic cell death [50].

The lysis of tumour cells in the context of the much-needed immune-stimulating signals provided by OVs can surpass the immune-suppressive microenvironment of a tumour and efficiently trigger an immune response [53]. Locally, OVs induce the production of various chemokines that attract immune cells. It has been shown in murine models that OV vaccination recruits and activates at the tumour site both antigen-presenting cells that prime the immune response, as well as natural killer and cytotoxic T cells that directly kill tumour cells [7,10]. OVs represent flexible therapeutic platforms that can be further manipulated in a number of ways to improve their anti-cancer vaccine potential. For example, not only are OVs immune-stimulatory themselves, but they can be engineered to encode immune-activating factors [54] like cytokines and chemokines [55,56,57], immune checkpoint inhibitor blocking antibodies [58,59], or even inhibitors of immunosuppressive factors [60]. One such example is a recombinant oncolytic HSV expressing 15-prostaglandin dehydrogenase, an enzyme that degrades the immunosuppressive signaling molecule prostaglandin E2, often overexpressed by tumour cells and tumour-infiltrating immune cells. This virus was shown to efficiently control tumour growth and metastasis by reducing the levels of peripheral myeloid-derived suppressor cells [61]. The combination of OVs and immune checkpoint blockade as also been shown to be a powerful vaccination strategy, particularly for poorly immunogenic tumours. For example, the combination of oncolytic New Castle disease virus with an anti-cytotoxic T-lymphocyte-associated protein 4 (CTLA)-4 blocking antibody resulted in over 60% cures in the murine B16F10 melanoma model and 100% protection against tumour rechallenge while either treatment alone was unable to cure any of the animals in this experimental setting [62]. In another study, the combination of Reovirus with PD1 blockade could efficiently control B16F10 tumours by a mechanism requiring both NK cells and CD8 T cells [63]. Finally, the combination of vaccinia virus with CTLA4 blockade in the Renca tumour model was shown to improve efficacy when immune checkpoint blocking antibodies were administered six days post-virus treatment [64]. Notably, the authors found that the antibody impaired viral replication if given earlier. These strategies and features of OVs, as well as the tools available to study the immune-modulatory facet of the therapy, have been thoroughly reviewed recently [53,65,66,67] and, thus, will not be further discussed here.

## 6. Clinical Evidence of OV-Induced Immunity

Clinical data on immune responses in OV-treated patients is quite limited at this time, with most immune data coming from animal models. Results from a phase I clinical trial using intravenously administered oncolytic reovirus for the treatment of advanced cancer patients showed that CD4 T cell, CD8 T cell and NK cell numbers were increased in 47.6%, 33%, and 28.6% of patients, respectively [68]. Another example is measles virus, for which increased intra-tumoural T cell infiltration, improved CD8/CD4 ratios and elevated serum levels of IFN-γ and other pro-inflammatory cytokines like IL-12 were observed post-treatment in cutaneous T cell lymphoma patients [69]. These data suggest that viral therapy can activate a potent immune response in patients, but does not identify whether it is directed towards the virus or the tumour. An important clinical consideration is the immune response directed towards the viral vector itself. For some viruses like reovirus, HSV, vaccinia, and measles virus, pre-existing neutralizing antibodies were found to be present in patients prior to treatment and the antibody titers increased following treatment [68,70,71,72,73,74,75]. Similarly, the immune profile observed in patients treated with CD40L-expressing replicating Ad showed that the treatment initially triggers a robust anti-viral immune response, which declines rapidly. However, the treatment also induced an anti-tumour immune response that was maintained for several weeks post-treatment [50]. A robust anti-viral immune response might actually be beneficial in facilitating a broader immune response towards tumour antigens by attracting and activating various immune cells to the tumour niche. Another clinical indication in line with OV-induced anti-tumor immunity comes from a phase III trial using the HSV T-Vec, for which patients with injectable melanoma lesions were treated intra-tumorally. For these patients, 15% of their non-injected and measurable lesions reduced in size by more than 50% following treatment, suggesting the development of an immune response upon treatment of distant lesions [76]. Finally, with the induction of anti-tumor immunity comes the risk of also inducing auto-immunity. Such a scenario was observed in an early clinical trial using intra-tumoural delivery of a GM-CSF-encoding vaccinia virus for the treatment of cutaneous melanoma [77]. During this study, the patients demonstrated the development of anti-viral and anti-tumoural immunity as well as the apparition of vitiligo, an autoimmune condition that results in the discoloration of the skin and is often observed in patients that develop an anti-melanoma immune response. With in-depth clinical evaluation of OV vaccines, our understanding of the interplay between anti-viral and anti-tumoural immunity will deepen and provide us with valuable information on how to best manipulate these responses to maximize therapeutic benefits.

## 7. Arming the Soldiers; TAA-Encoding OVs

OVs can be armed with tumour antigens to enhance the anti-cancer immune response. Although direct oncolysis on its own releases tumour antigens, OVs encoding TAAs provide an extra edge as transgene expression is initiated and amplified upon proficient viral infection within the confines of the tumour. Indeed, TAA-encoding OVs can efficiently trigger an immune response against poorly immunogenic antigens. A myriad of OV vaccines have been designed for a number of viruses and antigens. For example, an attenuated vesicular stomatitis virus (VSV) encoding dopachrome tautomerase (DCT) enhances both CD4 and CD8 antigen-specific T cell responses and improves VSV efficacy in the B16F10 murine melanoma model [78]. Vaccinia virus has also been extensively used to encode tumour antigens. For example, vaccinia expressing human carcinoembryonic antigen (CEA) in the context of an MC38 colon carcinoma model as well as vaccinia encoding oncofetal antigen 5T4 in the B16F10 and CT26 models have both demonstrated efficient development of a protective antigen-specific immune responses [79,80]. Several groups studied the use of a variant of measles virus encoding CEA for the treatment of prostate [81], glioblastoma [82], and ovarian [83] cancer and demonstrated delayed tumour progression and increased survival in mouse models. Although CEA can be used as a target for anti-cancer vaccination, these studies did not focus on the induction of anti-tumour immunity but rather used the transgene as a marker of viral gene expression. There is no study to our knowledge evaluating the potential of measles virus encoding a tumour antigen for vaccination purposes. An exhaustive list of the various OVs and antigens that were shown to provide therapeutic benefits is depicted in Table 1.

A variation on this idea is to doubly-arm a virus with a tumour antigen and an immune-stimulating factor. For example, Sillajen’s Pexa-Vec (formerly known as Jennerex’s JX-594) is an oncolytic vaccinia virus encoding the granulocyte-macrophage colony-stimulating factor (GM-CSF) and has demonstrated promising results as a single agent in both Phase I and II clinical trials for the treatment of hepatocellular carcinoma [84,85]. Accordingly, the double-engineering of a vaccinia virus to express both GM-CSF and the tumour-associated antigen HY was shown to increase systemic anti-tumour immunity and constituted an improved vaccination strategy compared to treatment with the parental virus [86]. As a final example, vaccinia virus encoding CEA together with three co-stimulatory molecules LFA-3, CD80, and ICAM-1, has been shown to generate even greater T cell stimulation compared to vaccinia-CEA in the MC38 murine colon carcinoma model [87]. As specific tumour antigens continue to be discovered, our repertoire of viruses encoding these antigens should correspondingly expand. This opportunity promises to effectively build up a bank of virus immunotherapies from which we can select depending on the mutational profile of each malignancy.

## 8. Two Is Better Than One; Heterologous Virus Prime-Boost for Anti-Cancer Vaccination

Viruses encoding TAAs are quite successful as monotherapies and, as such, their vaccination potential can only be further improved by combining these agents in prime-boost regimens. Initial exposure to an antigen of interest initiates an adaptive immune response, which can be further amplified upon subsequent boosting [88]. However, repeated administration of the same viral vaccine agent triggers a robust immune response directed towards the pathogen. Thus, the use of heterologous viral vectors encoding the same antigen as priming and boosting agents better directs the development of an immune response towards the encoded antigen rather than the vectors. When using viruses for such a strategy, the characteristics of each virus must be considered, as some will be better suited to prime the immune response, meanwhile others may be more appropriate as boosting agents. One promising priming candidate is Ad. Following administration of recombinant Ad, virally-encoded tumour antigens remain detectable for at least 32 days and stimulate a robust immune response that slowly declines over time [89]. This leaves a large window of opportunity to use a boosting agent and improve anti-tumour immunity. One such strategy that has been investigated is a heterologous virus prime-boost combination of Ad and the oncolytic Rhabdovirus Maraba MG1. Despite MG1-DCT being unable to mount an anti-DCT immune response when used as a priming agent, its use as a boosting vector following Ad-DCT priming significantly improved survival in the B16F10 melanoma model [90]. Another attenuated oncolytic Rhabdovirus, VSVΔ51, joins the ranks with MG1 as an excellent candidate boosting agent [78]. Both VSVΔ51 and MG1 are capable of boosting pre-existing T cell immune responses towards tumour antigens [78,90]. Furthermore, when used in the prime-boost regimens, the important anti-tumour immune response generated by these viruses reduces the impact of the anti-viral defense response, which could otherwise limit OV success [78,90]. Given the exciting pre-clinical data obtained using this strategy, a phase I/II clinical trial has commenced using a combination of Ad and MG1, both expressing the human cancer antigen MAGE-A3 (NCT02285816). More viruses and combinations have been tested in the prime-boost setting and are listed in Table 1.

## 9. Alternative Vaccination Strategies Using OVs; Reinventing the Wheel

### 9.1. Coating Viruses

As an alternative to encoding the antigens within the viral backbone, Cerullo and colleagues explored the possibility of directly coating the viral particles with antigenic peptides [113]. Their results showed that Ad dressed with tumour epitopes enhanced cytotoxic T cell activation and promoted cross-presentation by dendritic cells in both murine and human melanoma models. To do so, they used peptides with a poly-lysine tail extension to allow for anchoring to the membrane [113]. The ability to absorb the peptides at the viral surface was attributed to the negative charge of the adenoviral capsid. Notably, peptide-coated Ad demonstrated improved efficacy and induced stronger anti-tumour immunity compared to Ad administered with unconjugated peptides. However, the study did not investigate if this strategy is more efficient at inducing an immune response compared to viruses expressing the epitopes. The authors argue that their strategy bypasses several limitations of the classical method of encoding the tumor antigens directly into the virus and would be especially attractive and feasible as a personalized vaccine approach. Although it is technically possible to engineer OVs to encode patient-specific mutations, there is no report to date using this strategy. Furthermore, the engineering and pre-clinical testing of the safety of patient-tailored virus variants would be a time-consuming and costly approach that may limit the clinical feasibility of this approach. Using a single biotherapeutic agent that is further adapted for each patient, as is the case with peptide-coating of the virus, bypasses these limitations and is a more feasible approach. Whether the ability to absorb the peptides at the viral surface was attributed to the negative charge of the adenoviral capsid. Whether this approach can be generalized to other viruses remains to be explored.

### 9.2. cDNA Libraries

While common immunogenic tumour antigens are promising targets for anti-cancer vaccination, they are not necessarily expressed by all cancers and for some patients with high mutational burden, immunizing against their tumour mutanome constitutes a more suitable option. One of the most obvious hurdles for personalized anti-tumour vaccination strategies is the identification of novel tumour antigens that can be specifically targeted and confer protection without causing autoimmunity [114]. Following their identification, it is also important to determine which of these neo-antigens elicit the strongest and broadest immune responses and thus, are the best candidates for vaccination. For instances where the prediction and testing of immunogenic epitopes is not possible, perhaps the most plausible vaccination strategies could involve the use of cDNA libraries which surpasses this limitation. OVs encoding cDNA libraries constitute an original approach that was shown to confer therapeutic benefits. Indeed, the group of Richard Vile has shown that the immunization of mice with a library of VSV variants encoding human prostate cDNAs resulted in the rejection of established TRAMP-C2 tumours. Similar results were obtained using a VSV library generated from human melanoma cDNA for the treatment of murine melanoma [112]. Although as little as three intravenous administrations of the VSV-human prostate cDNA library could efficiently regress tumours, the escape tumour variants were completely refractory to further treatment using the same library. Interestingly, a second VSV library generated from cDNA from the escaped variants successfully provided protection against these immunoedited tumours [112]. Although the use of cDNA libraries encoded into OVs is an attractive strategy, there are risks of developing autoimmunity associated with such approach. While no autoimmunity was observed following vaccination with the VSV-cDNA library in this study, more work will be required to ascertain the safety of this approach.

### 9.3. Non-Replicating Viruses

Although OVs are typically known for their ability to hijack and replicate within host cells, OVs that have lost this capacity are still attractive therapeutic candidates given that they maintain their ability to specifically target a population of cells. Non-replicating viruses can be used in the context of cancer therapy as delivery vectors and potent immune stimulators. For example, non-replicating *Rhabdovirus* particles generated by low-dose irradiation are cytotoxic to leukemia cell lines and patient samples and elicit a tumour-specific immune response [115]. As suggested by the authors, this strategy could be a safer anti-tumour vaccination method for immune-compromised cancer patients. Although these results are promising, much higher doses of virus are required compared to the usual therapeutic dose administered to animals and one must take into consideration the manufacturing capacity and feasibility of producing such quantities of high-grade virus for clinical application.

### 9.4. Infected Cell Vaccines

A strategy that combines both the immune-stimulating capacity of the virus and expression of virally encoded factors by the infected tumour cell is the use of irradiated OV infected cells as vaccines. This treatment is a variation of the oncolysate approach pioneered in 1967 by Jean Lindenmann and Paul Klein. This initial work demonstrated that the vaccination of animals with homogenates obtained from tumor cells infected with oncolytic Influenza virus could provide protection against subsequent tumor-rechallenges [116]. Similarly, irradiated VSV infected L1210 leukemia cells elicit an anti-tumour immune response greater than that conferred by irradiated necrotic or apoptotic cells or inactivated virus alone [117,118]. A similar strategy used by Lemay et al. in the CT26 colon carcinoma model shows that vaccination with VSV-infected-irradiated tumour cells controls growth of subsequent homologous tumours in both subcutaneous and lung metastasis models [119]. Furthermore, even more impressive results were obtained when using a GM-CSF expressing virus. With this virus, maturation of dendritic cells and intratumoural infiltration by natural killer cells were both increased compared with the control virus-infected cells and resulted in correspondingly improved control of B16F10 lung tumours. Along similar lines, the infected cell vaccine approach has recently been demonstrated to induce anti-tumour immune responses using both reovirus [120] and an IL-12-expressing HSV [121], demonstrating that applicability of this strategy to several OV platforms.

## 10. The Barriers to Viral Vaccination Strategies

An important limitation to consider when exploiting OVs as platforms for vaccination is the unavoidable immune response generated towards the virus itself. This not only limits delivery, but also dampens the response towards tumour antigens. Under the selective pressure of an immune system that is efficient at eliminating pathogens, viruses have evolved mechanisms to avoid immune recognition. Of these, vaccinia virus encodes B18R, a viral protein that functions as a decoy receptor for type I IFNs and therefore prevents IFN-α and -β from mediating their anti-viral effects. To counteract this effect, the group of Steven Albelda engineered the virus to express IFN-β. Using a lung cancer model, they demonstrated improved induction of anti-tumor immunity and tumor infiltration by CD8 T cells following treatment with their IFN-β-expressing vaccinia variant compared to the control virus [122]. Following the same concept, a VSV variant expressing IFN-β demonstrated an improved capacity to induce systemic anti-tumor immunity in murine tumor models of non-small cell lung cancer [123]. Another example is HSV, which also evolved various mechanisms to avoid immune clearance. One of these involves the protein ICP47, which blocks the function of the transporter associated with antigen presentation and, therefore, impairs antigen presentation. Interestingly, an ICP47-deleted variant also over-expressing GM-CSF was able to control injected, as well as distant, tumors and demonstrated an improved capacity to induce anti-tumour immunity [124].

There is clinical evidence demonstrating the generation of an anti-viral immune response upon OV-treatment of patients [68,70,71,72,73,74,75]. For example, patients treated with an oncolytic Ad combined with low-dose temozolomide generated Ad capsid-specific neutralizing antibodies upon treatment [50]. Despite this, viral DNA was detectable up to 74 days post-treatment, suggesting effective viral replication. Disease control was achieved in 67% of the chemotherapy-refractory patients, demonstrating that OV-therapy can be effective even in the presence of anti-viral antibodies [50]. Anti-viral immunity also limits efficient multiple-dosing of the treatment and is even more of an obstacle for viruses against which the general population is already vaccinated, as is the case with measles and vaccinia viruses. The complement system, together with the neutralizing antibodies generated as a result of immunization, can severely impede virus delivery to the site of the tumour [125,126]. Several methods to overcome these limitations have been studied. Notably, the co-administration of complement inhibitors was shown to restore tumour delivery of vaccinia virus to levels comparable to the ones obtained for non-immune animals in a rat tumour model [126]. Another strategy that was proven efficient is to use cell carriers as Trojan horses in order to achieve stealth delivery of OVs [127,128]. Numerous cell types have been investigated for their ability to improve OV delivery, including tumour cells [129], monocytes [130], dendritic cells [131], T cells [132,133], mesenchymal stem cells [134], and insect cells [135]. While each of these cell types has its distinct advantages as OV delivery vehicles, the use of tumour cells is particularly appealing as this approach essentially mimics an infected cell vaccine and can, therefore, promote anti-tumour immune responses. Similarly, immune cell carriers may double as antigen presenting cells when infected with OVs expressing TAAs; however, studies investigating this are currently lacking. An alternative way to avoid immune recognition is to modify the appearance of the virus to the immune system. By exchanging the surface glycoprotein of VSV for that of the lymphocytic choriomeningitis virus, Muik and colleagues demonstrated that the natural neurotropism of the wild-type virus is reduced following intracranial administration [136]. This work demonstrates how pseudotyped virus can escape humoral immunity as a result of viral glycoprotein exchange [137].

## 11. Conclusions

Our knowledge of tumour immunology has significantly increased in recent years, pushing immunotherapy to the front lines of cancer treatment. With the successes of oncogenic virus immunization and the targeting of tumour antigens to treat established malignancies, anti-cancer vaccination strategies have proven their worth for translation towards the clinic. The increasing drive for effective immunotherapies is certainly demonstrated through the many clinical trials initiated for virus-based monotherapies, as well as prime-boost regimens using viral vaccine platforms. Based on their success to-date, the development of effective cancer vaccines should continue to improve outcomes for cancer patients and become a popular treatment option for otherwise difficult-to-treat disease forms. OV vaccination strategies may be the final “stab” needed to take cancer down.

## Figures and Tables

**Table 1:** TAA-encoding OVs used for anti-tumor vaccination.

Virus	Antigen	Tumor Model	Strategy
Ad	CEA	MC38	Boosting agent following DNA prime [91]
Ad	DCT	B16F10	Heterologous virus prime-boost [78,90]
Ad	gp33	B16F10	Heterologous virus prime-boost [92]
Ad	MART-1	NSFA-MART-1	Transduced dendritic cells as a prime [93]
Ad	PSA/PSCA	RM11-PSA	Stand alone priming agent [94,95]
HSV	PAP	TRAMP-C2	Stand alone treatment [96]
Maraba	DCT	B16F10	Boosting agent following Ad prime [90]
NDV	LacZ	CT26-LacZ	Stand alone treatment [97]
Semliki Forest	Ova	MOSE-Ova	Heterologous virus prime-boost [98]
Sindbis	E7	TC-1	Encoding HSP70 and E7 as a priming agent [99]
Sindbis	LacZ	CT26-LacZ	Replication defective virus for vaccination [100]
Vaccinia	5T4	B16F10, CT26	Stand alone treatment [80]
Vaccinia	CEA	MC38-CEA	Stand alone treatment [79], Heterologous virus prime-boost priming [101], Co administration with vaccinia-B7 [101], Encoding B7-1, ICAM-1 and LFA-3 [87,102]
Vaccinia	E6/E7	MRC-5, TC-1	Stand alone [103], Boosting agent following DNA priming [99], Encoding HSP70 and E7 as a boosting agent [99]
Vaccinia	EphA2	A549	Encoding Bi-specific T cell engager [104]
Vaccinia	gp33	B16F10	Heterologous virus prime-boost priming [92]
Vaccinia	HY	MB49	Encoding GM-CSF [86]
Vaccinia	Ova	MOSE-Ova	Heterologous virus prime-boost [98]
VSV	BRAF	B16F10	Stand alone treatment [105]
VSV	DCT	B16F10	Boosting agent following Ad prime [78]
VSV	E6/E7	TC-1	Stand alone treatment [106]
VSV	gp-100	B16F10-Ova	Stand alone treatment [107,108]
VSV	Ova	B16F10-Ova	Stand alone treatment [7,107,108,109], VSV-infected dendritic cells [78], VSV-infected B cells [92]
VSV	gp33	B16-F10-Ova	VSV-infected B cells as boosting agent [92]
VSV	various	B16F10	Clones isolated from a cDNA library. Antigens: HIF-2a, Sox-10, c-Myc, TYRP-1, N-Ras and Cyt-c [110,111,112]

Ad: adenovirus; BRAF: serine/threonine-protein kinase B-raf; CEA: carcinoembryonic antigen; DCT: dopachrome tautomerase; GM-CSF: granylocyte macrophage colony-stimulating factor; HSV: herpes simplex virus; ICAM-1: intercellular adhesion molecule-1; MART: melanoma antigen recognized by T cells; PAP: prostatic acid phosphatase; PSA: prostate specific antigen; PSCA: prostate stem cell antigen; VSV: vesicular stomatitis virus.
